# Soil bacterial quantification approaches coupling with relative abundances reflecting the changes of taxa

**DOI:** 10.1038/s41598-017-05260-w

**Published:** 2017-07-06

**Authors:** Zhaojing Zhang, Yuanyuan Qu, Shuzhen Li, Kai Feng, Shang Wang, Weiwei Cai, Yuting Liang, Hui Li, Meiying Xu, Huaqun Yin, Ye Deng

**Affiliations:** 1 State Key Laboratory of Industrial Ecology and Environmental Engineering (Ministry of Education, China), School of Environmental Science and Technology, Dalian University of Technology, Dalian, 116024 China; 2 Key Laboratory of Environmental Biotechnology, Research Center for Eco-Environmental Science, Chinese Academy of Sciences, Beijing, 100085 China; 3 State Key Laboratory of Urban Water Resource and Environment, Harbin Institute of Technology (SKLUWRE, HIT), Harbin, 150090 China; 4 State Key Laboratory of Soil and Sustainable Agriculture, Institute of Soil Science, Chinese Academy of Sciences, Nanjing, 210008 China; 5 State Key Laboratory of Forest and Soil Ecology, Institute of Applied Ecology, Chinese Academy of Sciences, Shenyang, 110016 China; 6 State Key Laboratory of Applied Microbiology Southern China, Guangdong Institute of Microbiology, Guangzhou, 510070 China; 7 School of Minerals Processing and Bioengineering, Central South University, Changsha, 410083 China; 8 College of Resources and Environment, University of Chinese Academy of Sciences, Beijing, 100190 China

## Abstract

Understanding the abundance change of certain bacterial taxa is quite important for the study of soil microbiology. However, the observed differences of relative abundances by high-throughput techniques may not accurately reflect those of the actual taxon abundances. This study investigated whether soil microbial abundances coupling with microbial quantities can be more informative in describing the microbial population distribution under different locations. We analyzed relative abundances of the major species in soil microbial communities from Beijing and Tibet grasslands by using 16 S rRNA high-throughput sequencing technique, and quantified the absolute bacterial cell numbers directly or indirectly by multiple culture-independent measurements, including adenosine tri-phosphate (ATP), flow cytometry (FCM), quantitative real-time PCR (qPCR), phospholipid fatty acids (PLFA) and microbial biomass Carbon (MBC). By comparison of the relative abundance and the estimated absolute abundances (EAA) of the major components in soil microbial communities, several dominant phyla, including Actinobacteria, Bacteroidetes, Verrucomicrobia, Chloroflexi, Gemmatimonates and Planctomycetes, showed significantly different trends. These results indicated that the change in EAA might be more informative in describing the dynamics of a population in a community. Further studies of soil microbes should combine the quantification and relative abundances of the microbial communities for the comparisons among various locations.

## Introduction

Microorganisms play essential roles in key biogeochemical cycles in almost all environments^1^. Particularly in soil, the microbial community with high taxonomic diversity and metabolic potential, can serve as a great biological indicator to reflect the major ecological processes^2, 3^. Therefore, to measure the abundance of some key populations is fairly important for understanding the contributions of the microbial community in soils^4^. However, obtaining accurate population density of key taxon is not an easy task. Often capturing individuals is an efficient mean available for macroscopic community surveys^5^, but rarely are all individuals in microbial community identified^6, 7^. With the rapid development of high-throughput molecular technologies, such as Illumina MiSeq/Hiseq sequencing, the relative abundances can be easily obtained^4, 8–11^. However, since relative abundance is expressed as a proportion of the total sample, the reliability of such estimates to reflect actual abundance of populations is insufficient^4, 12^.

Estimation of the absolute population abundances by combining the corresponding relative abundances with the absolute bacterial density, is an important prerequisite for the in-depth analysis of the structure and function of the microbial communities in natural environments^12^. The bacterial density is a fundamental measure in microbiology, but its assessment is often tedious^13^. To overcome this limitation, the rapid and reliable techniques are urgently needed to quantify the microbial populations in various environments, especially in soil with extremely high biodiversity^14^.

With the development of culture-independent measurements, various approaches, including direct counts of extracted bacteria, biochemical measurement and nucleic molecular approaches, have been used to quantify the microbial quantities^13, 15–18^. Recently, some studies have been done to compare these quantification methods. Flow cytometry (FCM) and fluorescence microscopy (EM) are two powerful direct counting methods, but the FCM is more rapid and accurate on enumerating bacterial cell numbers than visually observed EM^19^. Both methods use various fluorescent dyes to stain the bacterial cells, such as 4′,6-diamidino-2-phenylindole (DAPI), acridine orange (AO), propidium iodide (PI) and SYBR Green I (SYBR-I)^20–22^. Among of them, SYBR-I is the most commonly used fluorescent dye, because it is known to exhibit a high fluorescence quantum yield upon forming a complex with DNA molecules, and its background fluorescence is quite low^23^. Adenosine tri-phosphate (ATP), as the energy currency of all living cells, has been promoted as a potential indicator for viable quantities estimation for several decades^24–26^. The ATP measurement is fast, robust, and easy to perform, but it should be complemented with FCM or EM when it is used to estimate the bacterial cell numbers^13, 15^. In addition, quantitative real-time PCR (qPCR) with 16 S rRNA gene-based specific primers has been utilized as a sensitive and rapid method to quantify total bacterial community^27, 28^. The bacterial cell numbers measured by FCM, ATP, EM and qPCR, have been compared for diverse environments, such as drinking water^29^, wastewater^30^, activated sludge^19^, sediments^13^ and soil samples^17^. Almost all of these studies have proved that the FCM is more appropriate to monitor the cell numbers. Although both the FCM and ATP have been considered to be powerful measurements for the quantification of bacterial cell numbers, these two methods require a pre-suspension procedure of soil samples. Efficient detachment of bacterial cells is crucial for assessing microbial numbers^31^. However, there is no agreement on which procedure gives the best results with which type of substratum.

For the state of soil matrix, the soil microbial biomass, defining as the mass of the living component of soil organic matters, can be quantified by extracting a specific component of microbial biochemical substances. The classical method using chloroform fumigation to determine the microbial biomass carbon (MBC) was developed 40 years ago^32, 33^ but is still widely used today^34, 35^. Besides, the phospholipid fatty acids (PLFA) can be also served as biomarkers for bacteria in general, offering sensitive reproducible measurements for characterizing the numerically dominant portion of soil microbial community without cultivating the organisms^18, 36^. The PLFA analysis, based on modern biochemical techniques, shows many advantages over the conventional culturing methods, such as accuracy, quantitation, simple in operation, as well as no strict requirements for the microbial physiological status and sample conservation duration^16, 37^. However, the significant differences existing in those measurements, such as FCM and PLFA, make it difficult to compare the microbial quantities with the same criterion.

With the development of high-throughput nucleic molecular technologies and various quantitative measurements of microbial quantities, the relative abundances of microbial communities and absolute microbial quantities become easy to be estimated^13, 38^. Props *et al*. combined the high-throughput sequencing approach (16 S rRNA gene) with FCM to quantify the absolute taxon abundances on a cooling water system, which provided evidence that the increase in relative abundances could not necessarily relate to the increase in absolute abundances^39^. Stammler *et al*. demonstrated that combining the next-generation 16 S rRNA gene sequencing with qPCR could provide novel insights into the structure and the dynamics of intestinal microbiomes^40^. Smets *et al*. proposed a method with a simple addition of an internal standard at the DNA extraction step, to simultaneously measure the soil bacterial abundances and community composition via 16 S rRNA gene sequencing^41^. However, there is still a lack of systematic studies combining the absolute and relative measures to reveal the differences of microbial communities in diverse soil ecosystems. Besides, soils with distinct climates were good to identify major comparative consequences of the microbial communities^42^.

In the present study, distinct grassland soils from Tibet plateau and Songshan mountain in Beijing were collected which were over thousands of miles away. The relative abundances of microbial communities were analyzed by sequencing microbial 16 S rRNA gene amplicons using Illumina MiSeq technology. Diverse measurements, including ATP, FCM, qPCR, PLFA and MBC, were systematically compared and applied to quantify the total numbers of all individuals. Our results suggested that the relative abundances of species is insufficient to accurately figure out the absolute abundances of those in the studies of microbial communities, thus the quantification analysis of the bacterial absolute abundances is essential for the comprehensive analyses of the microbial community.

## Results

### Relative abundance of species in grassland soils from Beijing and Tibet

A total of 1,118,928 paired-end sequences were obtained from 20 samples of these two sampling sites, with 24,931-83,244 sequences per sample, and the sequencing depth proved to be close to saturation (Supplementary Fig. S1). Finally, random re-sampling was performed with 24,931 sequences per sample, resulting in 498,620 OTUs at 97% identity level.

**Figure 1:**
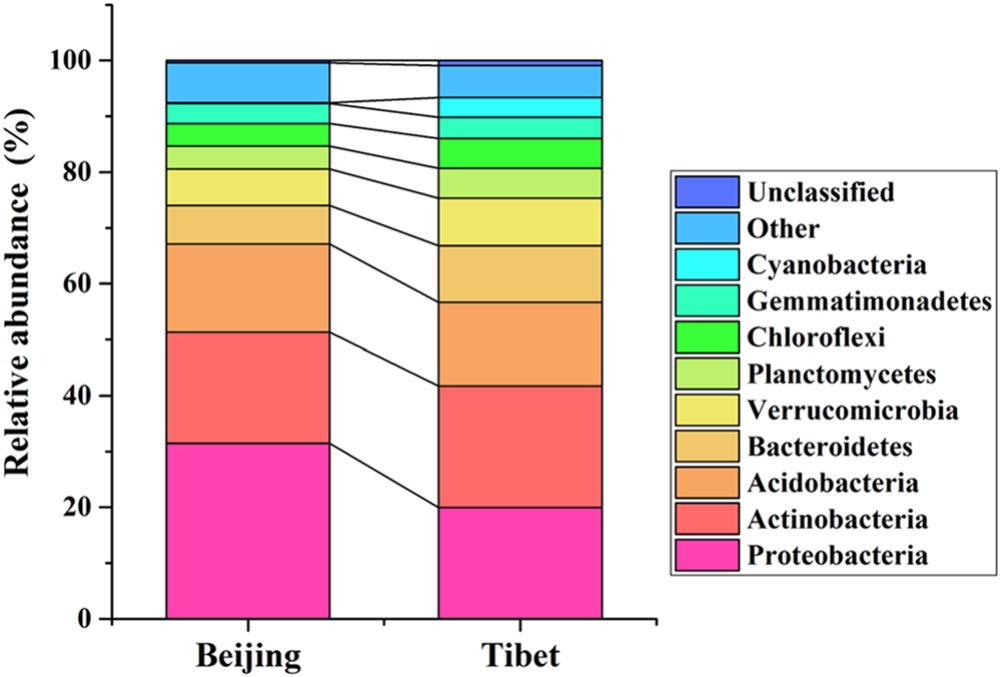
Relative abundances of nine dominant phyla in two sampling sites.

**Figure 2:**
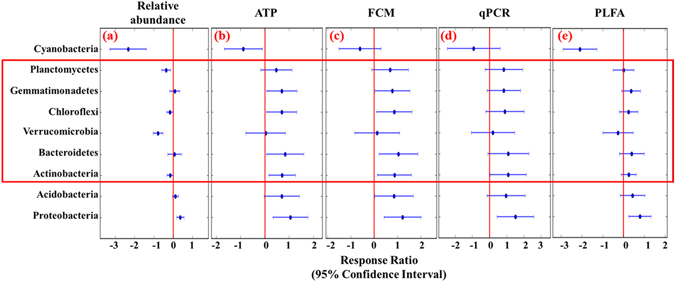
Response ratio for the differences between the relative abundances and estimated absolute abundances (EAA) of the major phyla in Beijing and Tibet grasslands. Significance was determined using the response ratio analysis at a 95% confidence level. The 95% CI of a response variable without overlapping with zero represent a significant result, otherwise, with non-significance. The response ratio more than zero means the abundances of Beijing more than Tibet samples, and vice versa.

The detrended correspondence analysis (DCA) showed that the structure of the microbial communities was significantly different in the two sampling sites, which has been confirmed by all three non-parametric dissimilarity tests (MRPP, ANOSIM, and ADONIS) (Supplementary Fig. S2, Table S2). Further comparison of the microbial taxonomic composition at the phylum level showed that 24 out of 46 phyla were shared by all samples, of which nine phyla represented over 90% of sequences in each sample (Fig. 1). Additionally, the composition of these nine dominant phyla differed significantly (P < 0.05) between Beijing and Tibet samples (Supplementary Table S2). The Proteobacteria and Actinobacteria were the most abundant bacterial phyla in both Beijing and Tibet grasslands (Fig. 1). The other seven phyla dominated in both sites, including Acidobacteria, Bacteroidetes, Verrucomicrobia, Planctomycetes, Chloroflexi, Gemmatimonadetes, and Cyanobacteria. Response ratio analyses at 95% confidence interval (CI) were performed on the relative abundances and absolute abundances of the major phyla between those two sampling sites (Fig. 2a, Supplementary Fig. S3a). For the relative abundances of the major phyla, the phylum Proteobacteria in Beijing was significantly higher than that in Tibet, while the phyla Verrucomicrobia, Planctomycetes, and Cyanobacteria were significantly lower than those in Tibet (Fig. 2a). Yet other phyla were not remarkably different.

### Absolute quantification of microbial quantities by multiple measurements

**Figure 3:**
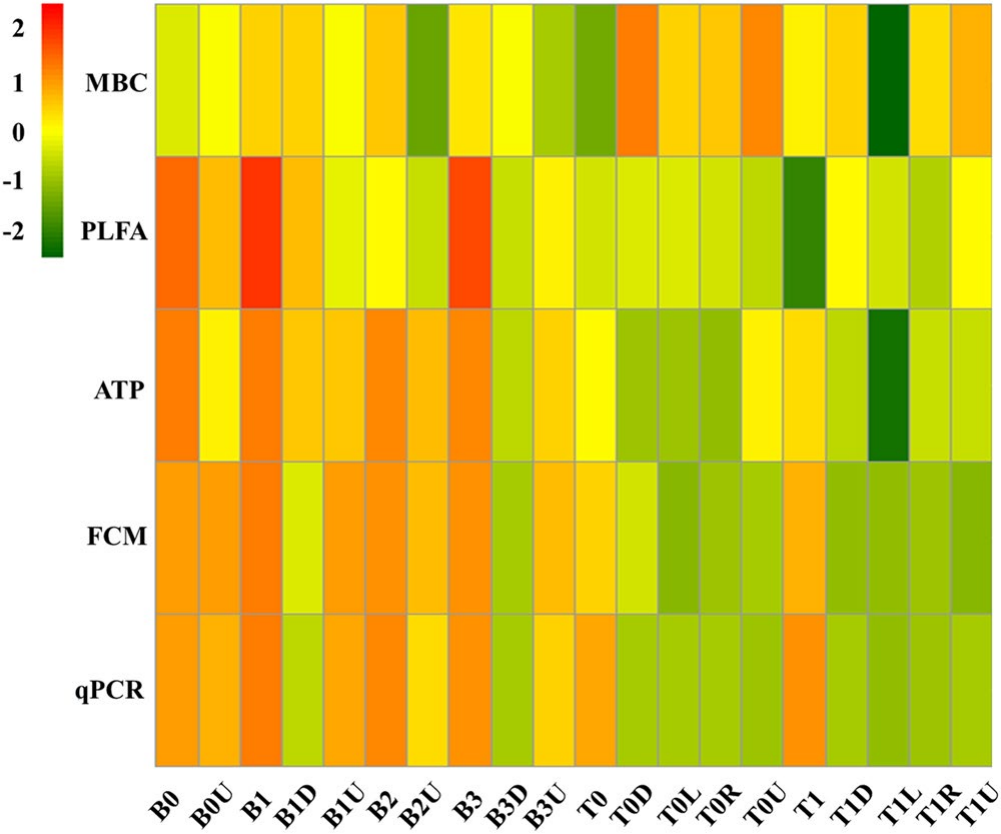
Comparison of the bacterial cell numbers derived from different measurements. Differences were visualized by heatmap. FCM: flow cytometry, ATP: adenosine tri-phosphate, qPCR: quantitative PCR, PLFA: phospholipid fatty acids, MBC: microbial biomass Carbon.

The absolute microbial biomass quantified by PLFA and MBC methods were 16.4 ± 10.75 nmol and 213.02 ± 164.56 μg per gram of dry soil, respectively (Table 1). Based on an empirical conversion that 1.40 × 10^–8^ nmol bacterial PLFA cell^−1^ approximately equal to 2.0 × 10^−5^ μg bacterial C per cell^18^, the bacterial cell numbers derived from bacterial PLFA concentrations was an average of 1.17 × 10^9^ ± 7.68 × 10^8^ cells g^−1^ dry soil, as well an average of 1.10 × 10^10^ ± 8.20 × 10^9^ cells g^−1^ dry soil was derived from the MBC concentrations. The absolute bacterial cell numbers directly determined by FCM averaged 9.47 × 10^8^ ± 1.07 × 10^9^ cells g^−1^ dry soil, whereas averages of 4.98 × 10^8^ ± 4.53 × 10^8^, and 3.90 × 10^9^ ± 5.24 × 10^9^ cells g^−1^ dry soil were derived from ATP and qPCR methods respectively, after some appropriate transformations (Table 1). All the tests of ATP, FCM, qPCR and PLFA quantification methods showed a good repeatability, but the MBC method showed obvious inconsistency with others (Fig. 3, Supplementary Table S3). Except MBC, the cell abundances based on FCM and qPCR showed greater variations within 10 replicates than the cell numbers obtained by ATP and PLFA assay between methods (Supplementary Fig. S4).

**Table 1:** Absolute quantification of microbial biomass in the soil samples determined by different measurements.

	ATP (cells g^−1^ dry soil)		FCM (cells g^−1^ dry soil)		qPCR (copies g^−1^ dry soil)		PLFA (cells g^−1^ dry soil)		MBC (cells g^−1^ dry soil)	
	Mean	*SD*	Mean	*SD*	Mean	*SD*	Mean	*SD*	Mean	*SD*
All	4.98 × 10^8^	4.53 × 10^8^	9.47 × 10^8^	1.07 × 10^9^	3.90 × 10^9^	5.24 × 10^9^	1.04 × 10^9^	5.27 × 10^8^	1.07 × 10^10^	8.23 × 10^9^
Beijing	8.03 × 10^8^	4.54 × 10^8^	1.65 × 10^9^	1.07 × 10^9^	6.18 × 10^9^	5.88 × 10^9^	1.27 × 10^9^	6.49 × 10^8^	7.56 × 10^9^	3.96 × 10^9^
Tibet	1.93 × 10^8^	1.42 × 10^8^	2.47 × 10^8^	4.20 × 10^8^	1.62 × 10^9^	3.43 × 10^9^	8.06 × 10^8^	2.09 × 10^8^	1.37 × 10^10^	1.03 × 10^10^

Correlation analyses, including Pearson correlation, Spearman and Kendall rank correlation showed that the measurements from ATP, FCM, qPCR, and PLFA were significantly (*P* < 0.05) positive correlations with each other, however, all of them showed negative correlation with MBC (Table 2, Supplementary Table S3).

**Table 2:** Pearson correlations (r values, n = 20) between different measurements. (**P < 0.01).

Pearson correlation	ATP	FCM	qPCR	PLFA	MBC
ATP	1	0.871**	0.827**	0.799**	−0.16
FCM		1	0.911**	0.720**	−0.257
qPCR			1	0.599**	−0.163
PLFA				1	−0.076
MBC					1

### Microbial quantities in different locations

**Figure 4:**
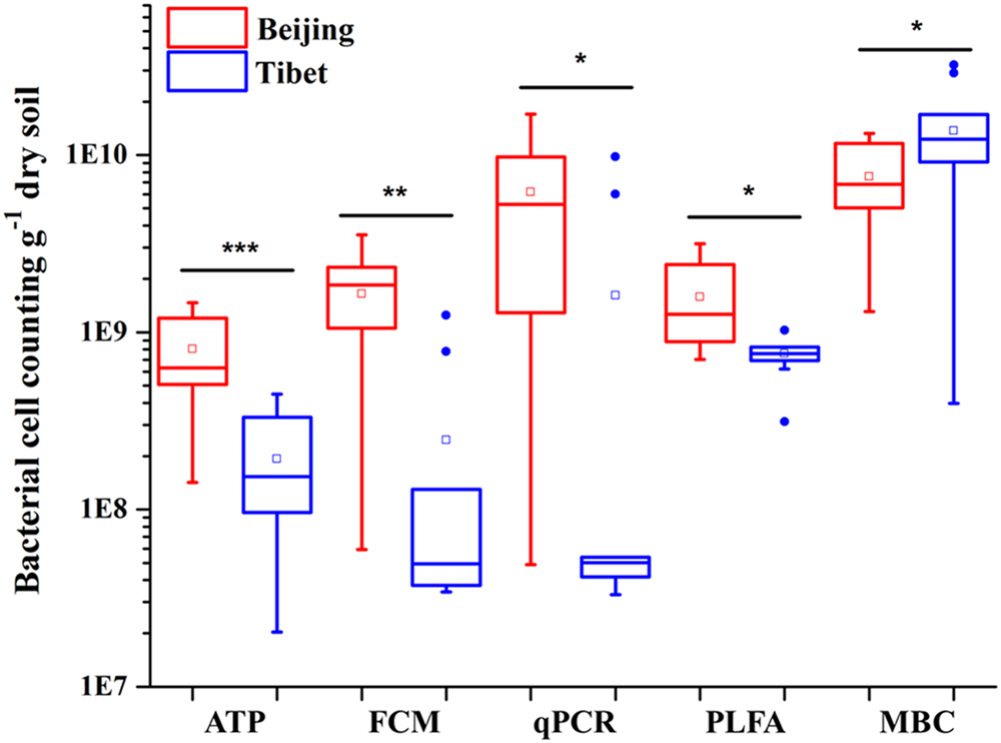
Comparison of the bacterial cell numbers between two sampling sites. (*P < 0.05; **P < 0.01; ***P < 0.001). Horizontal lines show median values, □ indicate mean values, boxes denote values comprised within the lower and upper quartile of the data, the vertical lines represent ranges, and • indicate outliers.

Different measurements showed that the microbial quantities were significantly different between the two sampling sites (Table 1, Fig. 4). Bacterial cell numbers based on ATP measurement, were significantly higher (*P* < 0.001) in Beijing sampling site (8.03 × 10^8^ ± 4.54 × 10^8^ g^−1^ dry soil, Beijing) than those in Tibet (1.93 × 10^8^ ± 1.42 × 10^8^ g^−1^ dry soil), similar with the results from FCM, qPCR and PLFA methods. However, the cell numbers derived from MBC were shown significantly lower in Beijing (7.56 × 10^9^ ± 3.96 × 10^9^ g^−1^ dry soil) than those in Tibet (1.37 × 10^10^ ± 1.03 × 10^10^ g^−1^ dry soil), which was not consistent with other methods. Besides, the Pearson correlation tests showed that all of these measurements, except for MBC, were significantly correlated with the environmental factors (soil moisture and TOC) (Supplementary Table S4). Thus the MBC measurement result was excluded from the following calculations.

### EAA of major phyla and classes in Beijing and Tibet sampling sites

**Figure 5:**
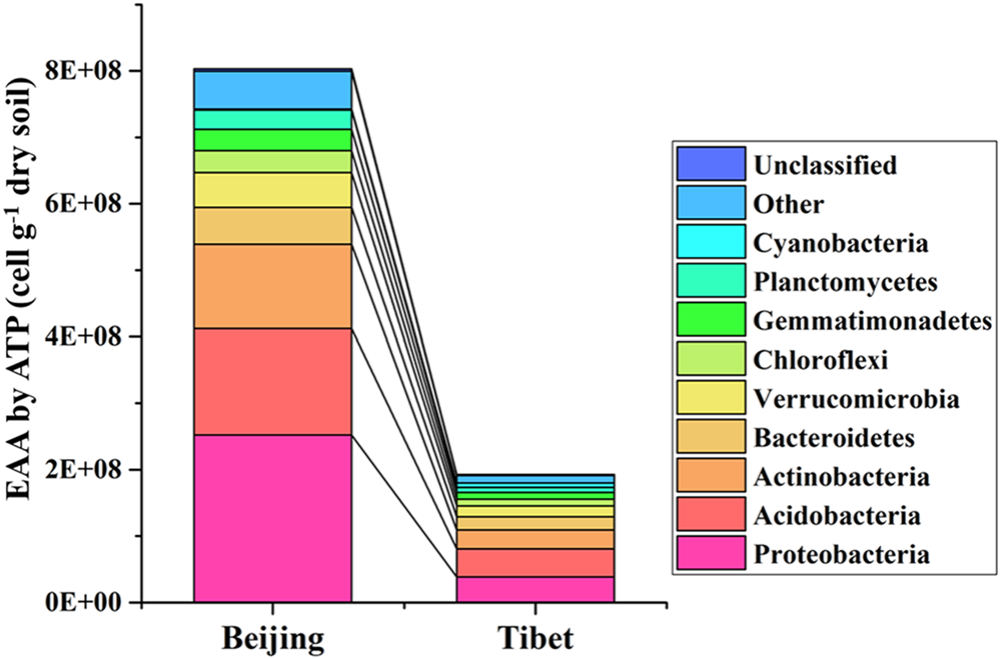
Comparison of the estimated absolute abundances (EAA) of the major phyla based on the ATP measurement in the two sampling sites. ATP: adenosine tri-phosphate.

We combined the bacterial quantities measured by the four different methods (ATP, FCM, qPCR and PLFA) with the relative abundances of those dominant phyla. Since the absolute microbial cell numbers in Beijing is significantly higher than that in Tibet, the estimated absolute abundances (EAA) of the major phyla showed distinct trends with the corresponding relative abundances, for example, by using ATP measured (Figs 1 and 5). Besides, the other three approaches, including FCM, qPCR and PLFA, showed very consistent trends with ATP measurement in different phyla (Fig. 2b–e). For Proteobacteria, both the relative abundances and EAA showed significantly higher in Beijing than those in Tibet. In addition, the Alphaproteobacteria, Betaproteobacteria, Gammaproteobacteria, and Deltaproteobacteria classes showed similar result that the relative abundances and EAA in Beijing were significantly higher than those in Tibet (Supplementary Fig. S3). However, dramatic changes were found in the EAA of some major phyla than their relative abundances. For example, the relative abundances of the phyla Actinobacteria, Bacteroidetes, Chloroflexi and Gemmatimonates, in Beijing samples were no significant difference with those in Tibet, but the EAA of those phyla were significantly higher in Beijing than those in Tibet; additionally, the phyla Verrucomicrobia and Planctomycetes, of which the relative abundances in Beijing were significantly higher than those in Tibet, showed no distinct difference in the EAA in Beijing and Tibet (Fig. 2).

## Discussions

**Figure 6:**
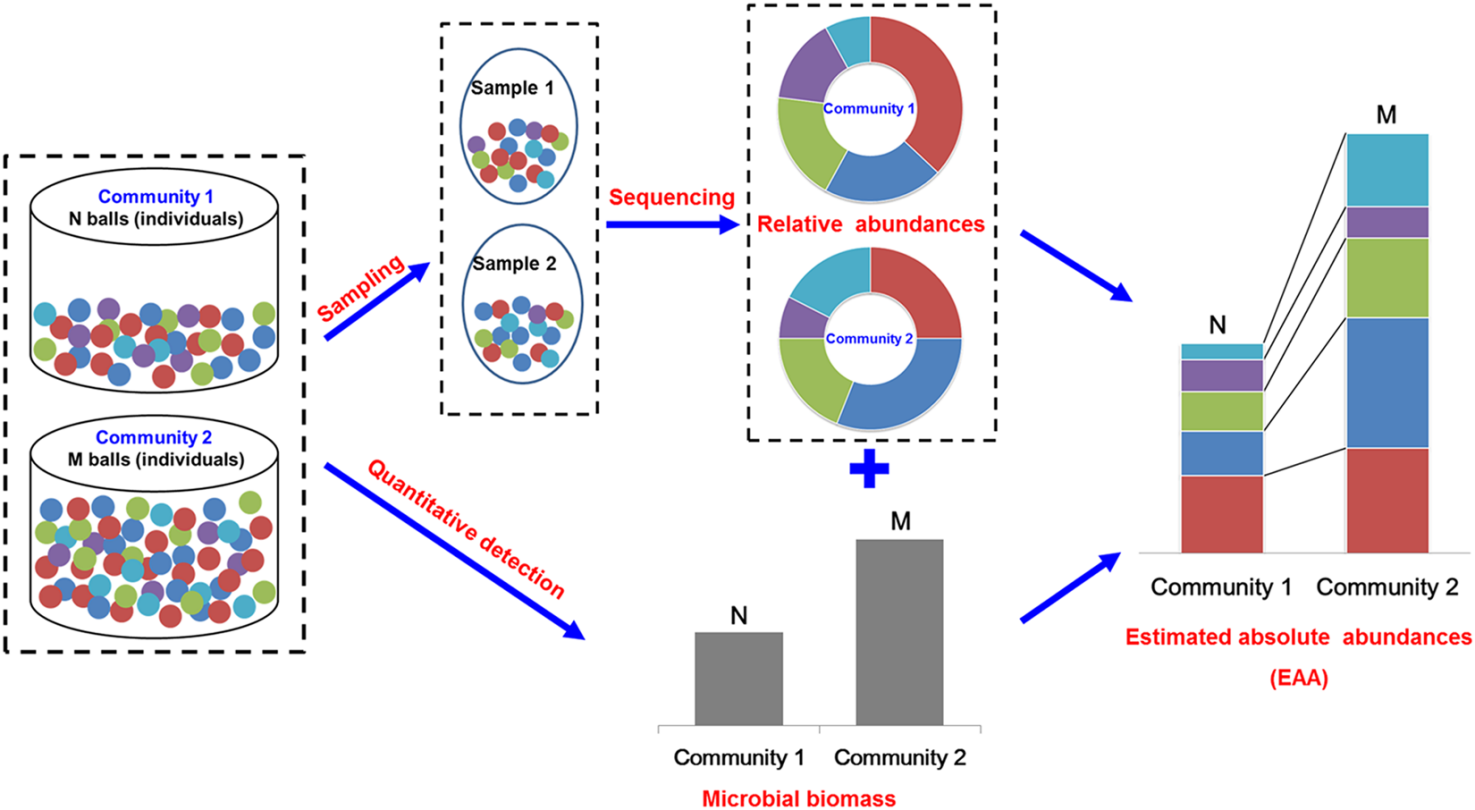
Conceptual model of the relationship between the estimated absolute abundances (EAA) with the relative abundances and the quantitative detection. The total number of balls in each community is quantified as N balls in community 1 and M balls in community 2, where N is about half of M. Meanwhile, the relative abundance of each color of ball is also calculated by regarding each community as an integral whole. The EAA could be obtained by combining the quantitative detection and the relative abundances.

As same as the macro-ecology, the differences of relative abundances could be inappropriate to interpret the real difference in abundances of microbial community in natural conditions^4^. In this study, we measured the EAA of the compositions in soil microbial communities by combining the corresponding relative abundances from the high-throughput sequencing approach with the absolute microbial quantities by various quantitative measurements. According to our results, we propose a conceptual model for the relationship between the EAA with the relative abundances and the quantitative detection (Fig. 6). For instance, in the given community 1 and 2, when we only considered the relative abundances of different taxa based on sampling, we could only obtain the composition of microbial community relatively, but when those compositions have been integrated with the quantitative estimation, the taxa abundance changes could be depicted more close to real abundance changes.

In soil matrices, bacteria are closely associated with various physical components, and thus some quantitative methods, like ATP and FCM assays, should suspend the bacterial cells as a pretreatment step. In the present study, a simple but efficient method was selected, which was previously described and calibrated for natural soil samples^17, 43^. While, as reported by Frossard *et al*.^13^, the bacterial cells were detached and separated from soil matrix particles by treatment with an ultrasonic probe, centrifugation in tubes receiving sample suspensions and Histodenz^®^ solution, which was also suitable for sediments and sludge samples. Although the detachment procedures were different, we both got a rough same amounts of cells for assessing the absolute bacterial quantities in natural soils. Some studies have discussed the detachment methods for the bacterial extraction in diverse environments, such as soils, sediments, activated sludge, and plant litter, however, the number of released bacterial cells could vary moderately among different detachment devices^19, 30, 31, 43^. Since the efficient detachment of bacterial cells is crucial for assessing bacterial cell numbers in natural environments, we have chosen the simple but quite stable way in this study and the final bacterial cell numbers obtained here are reasonable.

The absolute quantification of the microbial quantities is a crucial step for estimating absolute abundance of each taxon, where the accurate quantitative method should be evaluated key prerequisite. By comparison of bacterial cell numbers from total 20 soil samples, our results showed that the MBC would be unsuitable for this quantitative study (Table 2, Fig. 3), possibly because the soil microbial quantities measured by MBC were overestimated by fungi and protozoa^2, 44^. Except for the MBC method, bacterial cell numbers counted by the four quantitative methods, including ATP, FCM, qPCR and PLFA, showed a good repeatability and significant correlations (Fig. 3, Table 2). This observation showed that those four techniques focusing on the same biological component could provide conclusions with minor differences. The ATP, FCM and HPC methods have been applied to measure the microbial cells in drinking water and other aquatic environments, suggesting that the ATP analysis is a more rapid and easy tool for counting the intact microbial quantities^15^. Most studies comparing various quantitative measurements consider the FCM analysis as a most potential and robust single-cell enumeration technology for diverse environments, such as drinking water systems^29, 45^, cooling water system^39^, agricultural soil^17^, sediments^13^ and activated sludge^19, 30^. But it is possible that the FCM led to an underestimation of bacterial numbers, since in soil and sediment matrices bacteria are closely associated with physical components^17^. One study showed the estimation based on the ATP assay yielded significantly higher average bacterial abundances than the FCM method^13^. However, our study showed that the counting results from ATP assay were similar and significantly correlated (*P* < 0.001) with FCM (Tables 1 and 2). The reason for this difference may be due to the pretreatment step for FCM and ATP. Furthermore, the qPCR carried out with the same DNA with the amplicon sequencing analyses may be more appealing. A significant positive association was observed between FCM counts and qPCR quantification in the present study, whereas the absolute number detected by FCM was lower than qPCR method within 1–2 orders of magnitude, which was consistent with the study by Bressan *et al*.^17^. The difference between FCM and qPCR could be due to the combination of the inherent biases of all experimental steps, such as the efficiency of cell or DNA extraction, PCR inhibition due to the amplification efficiency and primer specificity, or underestimated counts of bacterial cells with FCM. In the previous study, qPCR relied upon total DNA extraction and could overestimate the bacterial cell numbers, since the exact number of copies of the 16 S rRNA gene in any given bacterial species varies^28, 46^. Few studies have compared PLFA measurements with the ATP, FCM and qPCR methods in the estimation of absolute microbial quantities. Our results from the biomarker PLFA for bacteria showed significantly correlated with the other three methods, and lower variation than that from qPCR. The PLFA profile is widely used to measure microbial biomass and community composition in soil and other types of environmental samples, but PLFA analysis is somewhat slow and expensive to carry out^47^.

According to the results of those four measurements, the soil microbial quantities appeared significantly higher in Beijing than those in Tibet. The correlation tests showed that the measurements, including ATP, FCM, qPCR and PLFA, were significantly correlated with the soil moisture and TOC (Supplementary Table S4). Soil moisture plays an important role for the terrestrial ecosystem processes, and also could determine the diversity of soil microbial communities^48–50^. We found that the moistures in Beijing were significantly higher than those in Tibet, which might lead to the higher bacterial cell numbers in Beijing sampling sites. On the other hand, the significantly higher of TOC, which might provide more substrates for the growth of microorganisms, also resulted in the higher the bacterial quantities in Beijing than those in Tibet^51^.

The most important question we tried to address is whether the changes in the absolute abundances of the components in microbial communities are inconformity with the shifts in relative abundances. Since the relative abundances of microbial community compositions was expressed as a proportion of the total sample, it had ignored the absolute abundances of species under different conditions^6^. To further verify the limitations of the relative abundances, we quantified the EEA of two distinct sampling sites by combining with the absolute microbial quantities, Beijing and Tibet. Several major phyla, including Actinobacteria, Bacteroidetes, Verrucomicrobia, Chloroflexi, Gemmatimonates and Planctomycetes, showed significant differences between the EAA with the relative abundances. For instance, the relative abundance of phylum Actinobacteria in Beijing is lower than that in Tibet with no significance, while the EEA of this phylum is significantly higher in Beijing and has significant correlation with moisture and TOC (Supplementary Table S5). It has been reported that the vast majority of Actinobacteria are important saprophytes capable of breaking down a wide range of plant and animal debris in the process of decomposition^52^. In addition, the phylum Proteobacteria, which has been recognized as a most dominant composition in soil microbial community^53^, shows significantly high in Beijing than Tibet, no matter relative abundances and EAA. Even though, from our result we are able to show that the quantification of the EEA for major phyla is essential and reliable, and has the potential to reflect the actual phenomenon of microbial communities in natural environments.

The conceptual model proposed in this study (Fig. 6) shows that the EEA of the different taxa change dramatically when the quantitative detection has been taken into account in the relative abundances. As described by Finnegan *et al*., any change in the relative abundance of a taxon could be explained in two ways^12^: (i) a change in its absolute abundance will obviously cause a corresponding change in its relative abundance; (ii) the shift in a taxon’s relative abundance may be driven by changes in the absolute abundance of other taxa in the assemblage, with little changes in the absolute abundance of the taxon itself. From the study of Fang *et al*., warming significantly altered the soil microbial community compositions, but did not significantly affect soil microbial biomass in all aggregate fractions^54^. For many ecological processes, the relative abundances of equivalent species on local and regional scales will not be influenced by changes in environmental conditions, while the fitness, such as soil quality, will only depend on the total microbial quantities^55, 56^. It has been reported that the microbial quantities could present sensitive respond to diverse environmental changes with decreasing in stress, such as heavy metal, or increasing in nutrient, including different fertilizations^57, 58^, while relative abundances of the microbial lineages showed distinct shifts^39^. Berga *et al*. demonstrated that with increasing salinity stress, the active bacterial community composition did not show significant changes directly, however, the bacterial abundances decreased significantly after the disturbance^59^. The possible reason might be that the change rates of the species in microbial communities showed distinct differences along with the microbial quantities responding to the environmental changes, resulting in that relative abundances of some species increase under stress, while others fall in nutrients. Therefore, understanding the ecological processes that shapes the absolute and relative abundances across the landscape and through time is ultimately required.

In summary, this study has demonstrated that the information reflected by the relative abundances could be insufficient to interpret the actual abundances, and the absolute bacterial quantities have been proved to be essential for the comprehensive analysis of the microbial communities in natural environment. Our study is also the first attempt to quantify the absolute abundances of the major components in the microbial communities by combining the relative abundances and bacterial quantities through diverse measurements.

## Material and Methods

### Site description and sample collection

Grassland soils from the Tibet plateau (29°36′21″N, 85°45′09″E) and the Songshan mountain located in Beijing (42°31′10″N, 115°49′32″E), China, were used in this study. At each site, we collected 10 parallel samples, each of which was homogeneously mixed by five surface soil cores (5–10 cm depth) within a 1 m*1 m quadrat. Soils were kept on ice until shipped to our laboratory for soil geochemical variables and microbial measurements.

The soils were all sieved by a 2 mm mesh, and the geochemical variables were measured as follows: pH was determined with a glass electrode in water-to-soil ratio of 2.5:1 (v/w). Soil moisture was measured by putting 2.0 g soil into 105 °C oven until constant weight was reached, and the percentage of weight loss after oven dry to the original weight was calculated as soil moisture content (%). The soil characteristics of the two sampling sites were summarized (Supplementary Table S1).

### Whole cell extraction from soil samples

The cell extraction was performed as described by Bressan *et al*. ^17^. Briefly, soil samples (5 g) were diluted in 45 mL of NaCl 0.85% solution (filtered at 0.22 μm) and homogenized by vortexing for 5 min at full speed. This suspension was centrifuged at 130 × *g* (gravities) for 5 min to exclude the large soil particle. The supernatant was then filtered by cell strainer (40 μm; BD FALCON), to remove particles prior to the further ATP and FCM analyses.

### Adenosine tri-phosphate (ATP) analysis

ATP was measured using the BacTiter-Glo^TM^ Microbial Cell Viability Assay (G8231, Promega Corporation, Dübendorf, CH) and a GloMax 20/20 Luminometer (Turner BioSystems, Sunnyvale, CA, USA). We applied an optimized protocol as described by Hammes *et al*.^15^, comparing with the manufacturer’s protocol for this product. Total ATP was measured as follows: 2 mL of the supernatant for each sample was transferred into a 2 mL Eppendorf tube and 50 μl of the ATP reagent was transferred to a separate sterile 1.5 mL reaction tube (Axygen). Both sample and reagent were heated for at least 1 min in a heating block at 38 °C. Then, 500 μl of the sample was transferred to the 50 μl reagent, and the mixture was incubated for a further 20 s in the heating block (38 °C). The luminescence was subsequently measured as an integral over 10 s, expressed in relative light units (RLU). The RLU values were converted to ATP concentrations using a calibration curve (R^2^ > 0.99), which was prepared with pure ATP standard (10 mM; Promega Corporation) diluted in ATP free, sterile water (Milli-Q) to different concentrations (10^−2^–10^4^ nM). A conversion factor of 1.75 × 10^−10^ nmol ATP per cell was applied to calculate the bacterial cell number^15^. All ATP measurements were done in triplicate, and all procedures were conducted under a sterile condition.

### Fluorescence staining and flow cytometry (FCM) analysis

All the soil supernatants (1 mL) with 10,000-time dilution were stained with 10 μL of SYBR^®^ Green I (100 × in DMSO; Invitrogen S7563), and then incubated for 15 min in the dark at room temperature before usage^30^. The flow cytometry (FCM) analyses were carried out on the BD FACSCalibur equipped with a 488 nm argon laser. Green fluorescence was collected in the FL1 channel at 600 nm, and red fluorescence was collected in the FL3 channel at 650 nm. All samples were collected as logarithmic signals and were triggered on the green fluorescence channel (FL1). The collection of data as FL1/FL3 dot plots allowed for optimal distinction between the stained microbial cells and instrument noise or sample background. In order to count the absolute number of bacteria in soil samples, the stained soil supernatants (450–700 μL) were added into the BD TruCount Absolut Count Tubes (BD Biosciences), and detected cell numbers after mixing. The FCM results were calculated according to the manufacturer’s protocol.

### Quantitative PCR (qPCR)

Total soil DNA was extracted from 0.5 g of moist sieved soil using the FastDNA^®^ SPIN Kit for Soil (MP-Biomedicals), following the manufacturer’s protocol. DNA was finally resuspended in 50 μL DES (DNase/Pyrogen-Free Water) and measured by Qubit^®^ 3.0 Fluorometer. Each PCR reaction mixture (20 μL) contained 10 ng of template DNA, 10 μL SuperReal PreMix Plus (SYBR Green, TIANGEN, FP205), and 1.5 μL of each primer (10 μM; Forward: 5’ -CCTACGGGAGGCAGCAG- 3′; Reverse:5′- TTACCGCGGCTGCTGGCAC- 3′). The qPCR reactions were performed in triplicate under thermal cycler conditions of 15 min at 95 °C, and 39 cycles of 10 s at 95 °C, 30 s at 55 °C and 32 s at 72 °C in a CFX Connect^TM^ Real-Time PCR Detection System (BioRad). All results were normalized and calculated using the ΔCt method.

### Phospholipid fatty acids (PLFA) analysis

Lipids were extracted from the soil samples using the modified Bligh-Dyer method^34^. Briefly, 2 g of each fresh soil sample was incubated in a 2:1:0.8 solution of methanol, chloroform, and phosphate buffer. The soil extracts were filtered and the chloroform phases collected. The phospholipids were separated from glycolipids and neutral lipids through silicic acid chromatography, subsequently saponified and methylated to fatty-acid methyl esters (FAME). FAME were then separated on a capillary gas chromatograph and identified using a gas chromatography/mass spectrometry. All procedures of PLFA analysis were performed in the Institute of Soil Science, Chinese Academy of Sciences (Nanjing, China).

A total of 109 different PLFA were detected and identified. Specific PLFA (15:0, i16:0, 17:0, i15:0, a15:0, i17:0, a17:0, cy17:0, 16: 1w5, 16: lw7t, 16:lw9, 18:lw7, and cy19:0), were used as biomarkers of bacterial origin, following the study of Frostegard^16^. The absolute bacterial biomass of the soils was calculated by adding the absolute content of each bacterial PLFA biomarkers. Bacterial cell numbers derived from bacterial PLFA concentrations are based on a conversion factor of 1.40 × 10^−8^ nmol per bacterial PLFA cell^18^.

### Microbial biomass Carbon (MBC) analysis

A rapid chloroform-fumigation extraction method was used to measure soil microbial biomass carbon (MBC) as previously described^60, 61^. Briefly, 15 g of each fresh soil sample was fumigated with alcohol-free CHCl_3_ for 24 h. Extraction for CHCl_3_-C was done with K_2_SO_4_ immediately after fumigation, and total microbial biomass Carbon with 10-fold dilution was detected by a MultiNC3100TOC analyzer. All procedures of MBC analysis were performed in the Institute of Applied Ecology, Chinese Academy of Sciences (Shenyang, China).

### Illumina sequencing analysis of 16 S rRNA gene amplicons

Microbial genomic DNA was extracted following the protocol of the FastDNA^®^ SPIN Kit for Soil. DNA quality and final concentrations were assessed by the ratios of 260 nm/280 nm, and 260 nm/230 nm using a NanoDrop Spectrophotometer (Nano-100, Aosheng Instrument Co Ltd.). All extracted DNA were stored at −80 °C for further amplifications. The primers 515 F (5′-GTGCCAGCMGCCGCGGTAA-3′) and 806 R (5′-GGACTACHVGGGTWTCTAAT-3′) targeting the V4 hypervariable regions of microbial 16 S rRNA genes were selected^62^, and tagged with paired barcode sequence (12 mer) for pooling of multiple samples in one sequencing run. All primers were synthesized by Sangon Biotech (Shanghai, China). Following amplification, the PCR product was used for agarose gel (1%) electrophoresis and purified by recovering the gel following the protocol of Gel Extraction Kit (D2500–02, OMEGA BioTek). From each sample, 150 ng of the PCR product, which was quantified by NanoDrop Spectrophotometer combining with Gel Image System (Taxon-1600), was collected and pooled with other samples. The sequencing run was conducted on MiSeq (Illumina) for 2 × 250 bp paired-end sequencing in Central South University (Changsha, China).

Raw data was processed using an in-house pipeline (http://mem.rcees.ac.cn:8080) which was built on the Galaxy platform but integrated various software tools related to sequencing processing. The operational taxonomic units (OTUs) were classified using UClust at 97% similarity level, and taxonomic annotations were assigned to each OTU’s representative sequence by the RDP 16 S Classifier^63^. Random resample was performed with the lowest sequences (>20,000 sequences) among samples. This resampled OTUs summary table was used for further various statistical analyses.

### Estimated absolute abundance (EAA) of each major taxon

We defined the estimated absolute abundance (EAA) as the product of a corresponding relative abundance multiplying with the total absolute microbial cell numbers. The relative abundance of this taxon was obtained from the 16 S rRNA sequencing analysis, while the absolute microbial cell numbers was quantified by the measurements mentioned previously.

### Statistical analysis

Non-parametric permutational multivariate analysis of variance of the Adonis function (ANONIS), analysis of similarities (ANOSIM), and multiresponse permutation procedure (MRPP) were used to evaluate the significant differences of the microbial community compositions between different sampling sites. Response ratio was performed to measure the relationship between the relative abundances and the EAA of major phyla^64^. The correlation between the bacterial cell numbers quantified by different measurements was conducted in SPSS22 using single-tailed Pearson correlation test, Kendall’s tau_b correlation, and Spearman correlation. Besides, the significance of difference between the microbial quantities from Beijing and Tibet was determined by a two-tailed T test.

## Electronic supplementary material

Supplementary information
