# Supplementary material for: Soil bacterial quantification approaches coupling with relative abundances reflecting the changes of taxa
Source: Sci Rep. 2017 Jul 6;7:4837. doi: 10.1038/s41598-017-05260-w (PMC5500469; doi:10.1038/s41598-017-05260-w)
Supplement: Supplementary file 1 — Supplementary information [file 41598_2017_5260_MOESM1_ESM.pdf]

Supplementary Information for *Scientific Reports*

Soil microbial quantification approaches coupling with relative abundances reflecting the changes of taxa in different locations

Zhaojing Zhang[1,2], Yuanyuan Qu[1,*], Shuzhen Li[1,2], Kai Feng[2], Shang Wang[2], Weiwei Cai[2,3], Yuting Liang[4], Hui Li[5], Meiying Xu[6], Huaqun Yin[7] & Ye Deng[2,8,*]

[1]State Key Laboratory of Industrial Ecology and Environmental Engineering (Ministry of Education, China), School of Environmental Science and Technology, Dalian University of Technology, Dalian 116024, China. [2]Key Laboratory of Environmental Biotechnology, Research Center for Eco-Environmental Science, Chinese Academy of Sciences, Beijing 100085, China. [3]State Key Laboratory of Urban Water Resource and Environment, Harbin Institute of Technology (SKLUWRE, HIT), Harbin 150090, China. [4]State Key Laboratory of Soil and Sustainable Agriculture, Institute of Soil Science, Chinese Academy of Sciences, Nanjing 210008, China. [5]State Key Laboratory of Forest and Soil Ecology, Institute of Applied Ecology, Chinese Academy of Sciences, Shenyang 110016, China. [6]State Key Laboratory of Applied Microbiology Southern China，Guangdong Institute of Microbiology, Guangzhou 510070, China. [7]School of Minerals Processing and Bioengineering, Central South University, Changsha 410083, China. [8]College of Resources and Environment, University of Chinese Academy of Sciences, Beijing 100190, China.

[*]Correspondence and requests for materials should be addressed to Y. D. (email: yedeng@rcees.ac.cn) or Y. Q. (email: qyy@dlut.edu.cn)

Ye Deng, Key Laboratory of Environmental Biotechnology, Research Center for Eco-Environmental Science, Chinese Academy of Sciences, Beijing 100085, China. Tel, +86-10-62840082.

Yuanyuan Qu, State Key Laboratory of Industrial Ecology and Environmental Engineering (Ministry of Education, China), School of Environmental Science and Technology, Dalian University of Technology, Dalian 116024, China. Tel, +86-411-84706250.

Supplementary Table S1. Summary of soil characteristics of the two sampling sites in this study.

Location	Elevation (m)	Latitude	Soil pH[a]	Soil moisture (%)	Total carbon content (TOC, mg/g)
Beijing	810-1349	40°31'10"N 115°49'32"E	6.17±0.48	21.49±7.35	46.69±27.96
Tibet	5088-5116	29°36'21"N 85°45'09"E	6.53±0.22	1.50±0.35	26.28±5.27

[a] Average individual soil variables (pH, moisture, and TOC) over 10 samples.

Supplementary Table S2. Dissimilarity tests of microbial communities from two sampling sites [a].

Dissimilarity	MRPP[b]		ANOSIM[c]		ADONIS[d]	
Beijing vs. Tibet	δ	P	R	P	F	P
Total	0.715	**0.002**	0.288	**0.002**	0.14	**0.001**
Proteobacteria	0.708	**0.003**	0.269	**0.005**	0.139	**0.001**
Actinobacteria	0.693	**0.003**	0.233	**0.007**	0.122	**0.003**
Acidobacteria	0.713	**0.002**	0.256	**0.002**	0.139	**0.002**
Bacteroidetes	0.755	**<0.001**	0.347	**0.001**	0.146	**0.002**
Verrucomicrobia	0.698	**<0.001**	0.318	**0.003**	0.16	**0.003**
Planctomycetes	0.798	**0.002**	0.379	**0.001**	0.125	**0.001**
Chloroflexi	0.72	**0.005**	0.271	**0.004**	0.139	**0.007**
Gemmatimonadetes	0.715	**<0.001**	0.269	**0.003**	0.148	**0.001**
Crenarchaeota	0.699	**0.002**	0.223	**0.01**	0.142	**0.004**
Cyanobacteria	0.866	**0.003**	0.213	**0.015**	0.109	**0.004**

[a]Different statistical approaches were used with Bray-Curtis distances, and P values were of corresponding significance tests.

[b]MRPP, multiresponse permutation procedure.

[c]ANOSIM, analysis of similarity.

[d]ADONIS, permutational multivariate analysis of variance with the Adonis function.

Supplementary Table S3. Spearman and Kendall rank correlation (r values, n=20) between different measurements.

Spearman correlation	ATP	FCM	qPCR	PLFA	MBC
ATP	1	**0.839****	**0.793****	**0.496****	-0.179
FCM		1	**0.854****	**0.518****	-0.245
qPCR			1	**0.504****	-0.181
PLFA				1	-0.142
MBC					1
**Kendall correlation**	**ATP**	**FCM**	**qPCR**	**PLFA**	**MBC**
ATP	1	**0.642****	**0.586****	**0.311***	-0.132
FCM		1	**0.670****	**0.364***	-0.111
qPCR			1	**0.402****	-0.085
PLFA				1	-0.085
MBC					1

*: $P < 0.05$; **: $P < 0.01$

Supplementary Table S4. Correlation tests (r values, n=20) between different measurements and the environmental factors.

Pearson correlation	Moisture	TOC
ATP	**0.806*** **	**0.708*** **
FCM	**0.803*** **	**0.697** **
qPCR	**0.679*** **	**0.748*** **
PLFA	**0.650** **	**0.626** **
MBC	-0.245	0.041

**: $P < 0.01$; *** : $P < 0.001$

Supplementary Table S5. Correlation tests (r values, n=20) between the environmental factors and the EAA of the major phyla based on the ATP measurement.

Pearson correlation	Moisture	TOC
Proteobacteria	**0.784****	**0.585****
Acidobacteria	**0.698****	**0.511***
Actinobacteria	**0.741****	**0.608****
Bacteroidetes	**0.575****	**0.528***
Verrucomicrobia	**0.595****	**0.508***
Chloroflexi	**0.665****	**0.512***
Gemmatimonadetes	**0.651****	0.433
Planctomycetes	**0.706****	**0.577****
Cyanobacteria	**-0.596****	-0.227

*: $P < 0.05$; **: $P < 0.01$

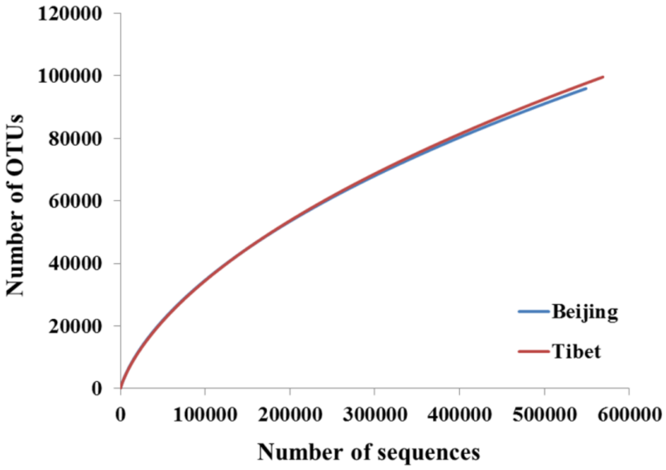

Supplementary Figure S1. Rarefaction curves based on 16S rRNA gene amplicon sequencing of soil microbial communities.

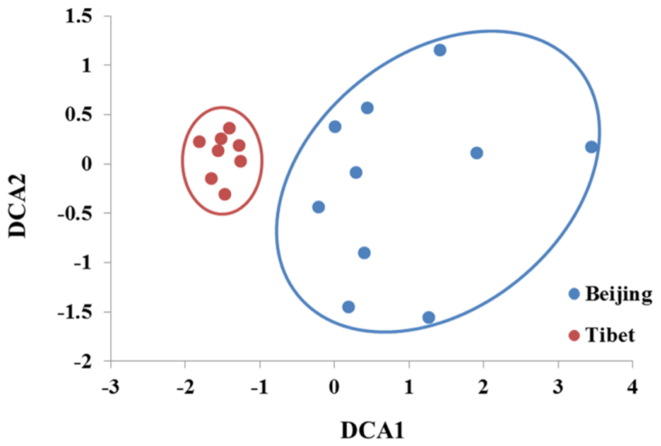

Supplementary Figure S2. Detrended correspondence analysis (DCA) of microbial community composition in Beijing and Tibet sampling sites based on 16S rRNA gene sequencing.

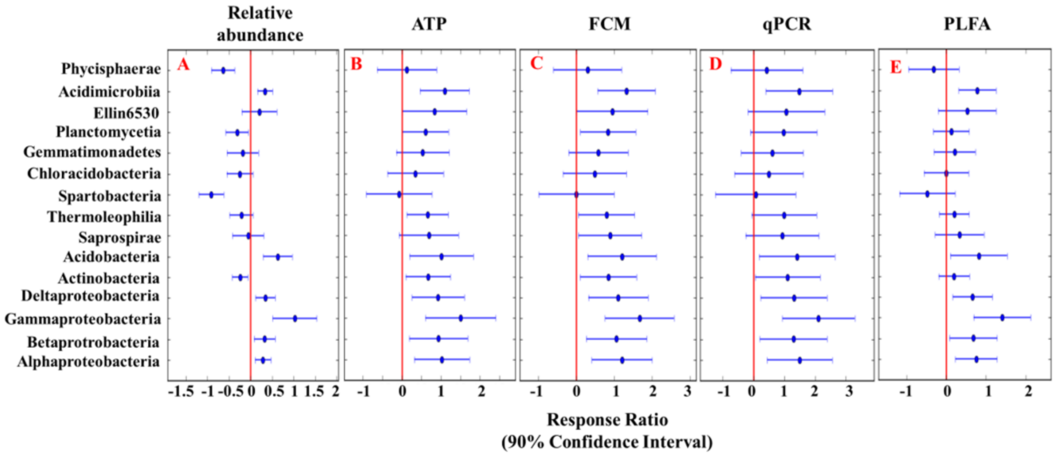

Supplementary Figure S3. Response ratio for the differences between the relative abundances and absolute abundances of the major class in the two sampling sites. Significance was determined using the response ratio analysis at a 95% confidence level. The 95% CI of a response variable without overlapping with zero represent a significant result, otherwise, with non-significance. The vertical solid line was drawn when the variables of Beijing samples equaled with those of Tibet samples.

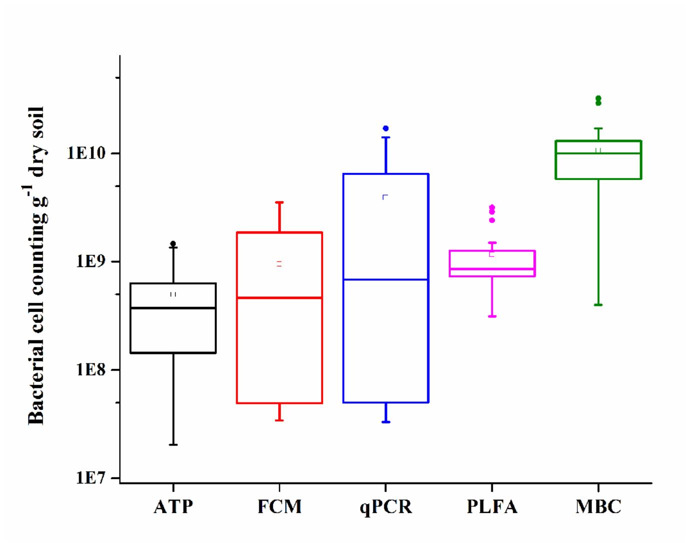

Supplementary Figure S4. Bacterial cell numbers assessed by five different methods in Beijing and Tibet sites. Horizontal lines show median values, □ indicate mean values, boxes denote values comprised within the lower and upper quartile of the data, the vertical lines represent ranges, and • indicate outliers.
